# A Lamellar Yolk–Shell Lithium‐Sulfur Battery Cathode Displaying Ultralong Cycling Life, High Rate Performance, and Temperature Tolerance

**DOI:** 10.1002/advs.202103517

**Published:** 2021-11-29

**Authors:** Jinyun Liu, Yingyi Ding, Zihan Shen, Huigang Zhang, Tianli Han, Yong Guan, Yangchao Tian, Paul V. Braun

**Affiliations:** ^1^ Key Laboratory of Functional Molecular Solids (Ministry of Education) Anhui Provincial Engineering Laboratory for New‐Energy Vehicle Battery Energy‐Storage Materials College of Chemistry and Materials Science Anhui Normal University Wuhu Anhui 241002 P. R. China; ^2^ National Laboratory of Solid State Microstructures College of Engineering and Applied Sciences Nanjing University Nanjing Jiangsu 210093 P. R. China; ^3^ National Synchrotron Radiation Laboratory University of Science and Technology of China Hefei Anhui 230026 P. R. China; ^4^ Department of Materials Science and Engineering Materials Research Laboratory Beckman Institute for Advanced Science and Technology University of Illinois at Urbana‐Champaign Urbana IL 61801 USA

**Keywords:** binding energy, cycling stability, nano‐CT, secondary batteries, shuttle effect

## Abstract

The shuttling behavior and slow conversion kinetics of the intermediate lithium polysulfides are the severe obstacles for the application of lithium‐sulfur (Li‐S) batteries over a wide temperature range. Here, an engineered lamellar yolk–shell structure of In_2_O_3_@void@carbon for the Li‐S battery cathode is developed for the first time to construct a powerful barrier that effectively inhibits the shuttling of polysulfides. On the basis of the unique nanochannel‐containing morphology, the continuous kinetic transformation of sulfur and polysulfides is confined in a stable framework, which is demonstrated by using X‐ray nanotomography. The constructed Li‐S battery exhibits a high cycling capability over 1000 cycles at 1.0 C with a capacity decay rate as low as 0.038% per cycle, good rate performance, and temperature tolerance at −10, 25, and 50 °C. A nondestructive in situ monitoring method of the interfacial reaction resistance in different cycling stages is proposed, which provides a new analysis perspective for the development of emerging electrochemical energy‐storage systems.

## Graphical abstract

An engineered lamellar yolk–shell structure of In_2_O_3_@void@carbon for the Li‐S battery cathode is developed for the first time to construct a powerful barrier that effectively inhibits the shuttling of polysulfides. The continuous kinetic transformation of sulfur and polysulfides is confined in a stable framework, which is demonstrated by using X‐ray nanotomography. The battery exhibits a long cycling life over 1000 cycles with a capacity fade rate as small as 0.038% per cycle, good rate performance, and temperature tolerance.

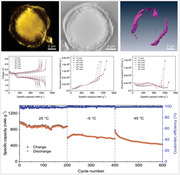

## 1 Introduction

Due to the ever‐increasing demands of global energy consumption, electrochemical energy‐storage systems are developing rapidly. Since that, high energy‐density secondary batteries with long cycle lives become increasingly important. Lithium‐sulfur (Li‐S) batteries are considered to be a promisingly candidate for next‐generation battery systems due to their high theoretical energy density of 2600 Wh kg^−1^ and capacity of 1675 mAh g^−1^,^[^ ^1^, ^2^, ^3^ ^]^ in addition to the high natural abundance of sulfur and low cost.^[^ ^4^, ^5^, ^6^ ^]^ However, practical applications of Li‐S batteries are hampered by the poor conductivity and large volume‐change of sulfur, the shuttle effect of soluble polysulfides, and slow redox reaction kinetic of sulfur.^[^ ^7^, ^8^, ^9^, ^10^, ^11^ ^]^

Considering the conductivity and volume‐change issues of the sulfur cathodes, engineering a yolk–shell structure with a reserved buffering space for sulfur is an efficient strategy. For example, Lin's group used electrospinning to synthesize the interconnected yolk–shell carbon nanospheres assembled fiber network, exhibiting a capacity of 700 mAh g^−1^ at 1.0 C after cycling 500 times.^[^ ^12^ ^]^ Liu and co‐workers prepared Fe_2_O_3_ nanoparticles within Mn_3_O_4_ nanosheet‐grafted hollow carbon capsules, which delivered a capacity as high as 1122 mAh g^−1^ after 200 cycles.^[^ ^13^ ^]^ Novel hierarchical yolk–shell microspheres composed of 1D bamboo‐like N‐doped carbon nanotubes (CNTs) encapsulating Co nanoparticle as sulfur host were reported by Park et al., which displayed a good stability.^[^ ^14^ ^]^ The yolk–shell structure has been considered to be able to alleviate several issues of Li‐S batteries; however, most of the studies focused on the properties at room temperature, which are quite different from the complicated temperature conditions in real applications.

Here, we report a novel lamellar yolk–shell indium oxide (In_2_O_3_)@void@carbon composite for encapsulating sulfur for the first time as a Li‐S battery cathode. The inner is assembled by dense In_2_O_3_ nanoflakes, forming numerous nanochannels, which exposes several sites to load sulfur and remain space for volume‐change; and the highly graphitized conductive carbon shell could improve the conductivity of the overall microsphere. A 3D space verified by X‐ray nanotomography (nano‐CT) proves an effective and continuous kinetic conversion of reversible capacity by limiting sulfur within the confined framework. As expected, an enhanced cycling performance is achievable at different temperatures by the accelerated redox reaction kinetics. The capacity decay behavior of the Li‐S battery at different temperatures is also demonstrated through the differential voltage (d*V*/d*Q*) analysis and a periodic galvanostatic intermittent titration technique (GITT). The adsorption models by density functional theory (DFT) calculations reveal the powerful capture ability of In_2_O_3_ toward polysulfides.

## 2 Results and Discussion

**Figure 1:**
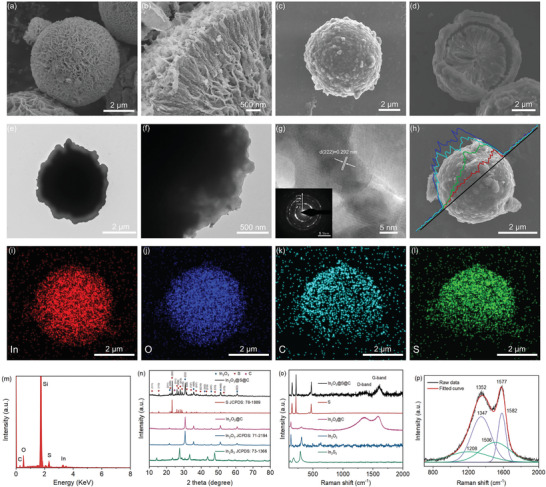
a) Low‐ and b) high‐magnification SEM images of the as‐synthesized In_2_O_3_ microsphere. c) SEM images of In_2_O_3_@C microsphere and d) sectional view of In_2_O_3_@C composites. e) TEM images of In_2_O_3_@S@C composites, f) edge of microsphere. g) HRTEM image inserted with a corresponding selected area electron diffraction pattern. h) Line‐scanning curves and i–l) elemental mapping images of the In_2_O_3_@S@C composites. m) EDS spectrum. n) XRD patterns of the In_2_S_3_, In_2_O_3_, In_2_O_3_@C, sulfur, and In_2_O_3_@S@C composites. o) Raman spectra and p) the deconvoluted Raman spectra from the Raman data in panel o) at the D and G band.

In_2_O_3_ microspheres were prepared by using a facile hydrothermal approach.^[^ ^15^ ^]^ Scanning electron microscopy (SEM) displays the morphology of a flower‐like spherical structure with numerous laminated nanoflakes assembled radially with a diameter of 6–8 µm (**Figure** **1**a,b). The formation mechanism of the structure was investigated through a series of synthesis under different conditions, as shown in Figure S1 (Supporting Information). In our study, cetyltrimethylammonium bromide (CTAB) was used as growth template, the microchannels among nanoflakes are correlated with the amount of CTAB. A higher reaction temperature makes the nanoflakes aggregate more tightly, and the overall shape tends to be spherical. Besides, both the amount of CATB and reaction temperature influence the assembly of nanoflakes on growth template. Figure S2c,d (Supporting Information) presents the SEM images of In_2_O_3_@SiO_2_. The In_2_O_3_@C yolk–shell structure after the carbon coating and alkali etching SiO_2_ process is shown in Figure 1c,d. The orange‐shaped nanochannel structure would provide several buffer spaces for the volume change of sulfur upon change–discharge. The hierarchical structure of In_2_O_3_@S@C microsphere is observed in Figure 1e,f and Figure S2e,f (Supporting Information). The zoomed‐in images of In_2_O_3_@C are shown in Figure S2h,i (Supporting Information). In addition, the high‐resolution transmission electron microscopy (HRTEM) image shows the highly crystalline nature by the clear lattice fringes with a *d*‐spacing of 0.292 nm, which matches the (222) plane of In_2_O_3_ (Figure 1g and inset). Elemental mappings and energy‐dispersive spectrometer (EDS) spectrum confirm the uniform distribution of In, O, C, and S in the In_2_O_3_@S@C microsphere (Figure 1h–m). The crystallographic structures of the as‐prepared In_2_O_3_ and In_2_O_3_@S@C are further identified by X‐ray diffraction (XRD) patterns (Figure 1n). All of the diffraction peaks are well‐indexed to the tetragonal In_2_O_3_ (JCPDS No. 71‐2194). For comparison, XRD pattern of the pure sulfur was also measured, which confirmed an obvious sulfur phase (JCPDS No. 78‐1889) within the In_2_O_3_@S@C composite.

The Raman spectrum of each sample is displayed in Figure 1o. The Raman spectra show two prominent peaks, which correspond to A_1g_ symmetry model of breathing vibration of rings (D‐band) and the E_2g_ phonon of sp^2^‐bonded hybridized carbon atoms (G‐band).^[^ ^16^ ^]^ The G and D peaks of the In_2_O_3_@C composite locate at 1577 and 1352 cm^−1^, respectively. The Raman spectrum was further fitted into four bands centered at 1582, 1506, 1347, and 1208 cm^−1^ by a Voigt model function (Figure 1p). The peaks at 1582 and 1347 cm^−1^ are the splitting peaks from peak G which are ascribed to the sp^2^ hybrid carbon atoms. The peaks at 1506 and 1208 cm^−1^ correspond to sp^3^ hybrid carbons belong to peak D.^[^ ^17^, ^18^ ^]^ The calculated peak area ratio of peak G and peak D is 1.32, indicating a high degree of graphitization and good conductivity. In addition, the high value of the ratio of *D*_FWHM_/*G*_FWHM_ shows better elastic and mechanical strength properties,^[^ ^19^ ^]^ which is beneficial for suppressing the volume change of sulfur during charge–discharge.

To compare the porous structure information between the In_2_O_3_@C and In_2_O_3_@S@C, N_2_ adsorption–desorption was measured (Figure S3, Supporting Information). The increase of the inner surface of the yolk–shell structure results in a high Brunauer−Emmett−Teller (BET) surface area of 81.1 m^2^ g^−1^. The BET surface area of In_2_O_3_@S@C decreases to 4.44 m^2^ g^−1^ after sulfur loading, which indicates the nearly complete filling of sulfur in the pores. The surface composition and chemical states of In_2_O_3_@S@C were characterized by using X‐ray photoelectron spectroscopy (XPS). Figure S4a (Supporting Information) displays the survey spectrum in which the S 2p_3/2_, S 2s, C 1s, In 3d_5/2_, In 3d_3/2_, and O 1s signals are observed.^[^ ^20^, ^21^ ^]^ The binding energies of In 3d_5/2_ and In 3d_3/2_ (Figure S4b, Supporting Information) are measured with peaks located at 444.5 and 452.0 eV, respectively, which indicate a trivalent oxidation state for In element.^[^ ^22^, ^23^ ^]^ As displayed in the O 1s spectrum (Figure S4c, Supporting Information), lattice oxygen peaks at 530.0, 531.9, and 532.5 eV correspond to —OH stems from In(OH)_3_, In—O, and C—O/C═O, respectively.^[^ ^24^ ^]^ Figure S4d (Supporting Information) shows the high‐resolution XPS spectrum of the C 1s. An asymmetric peak at 284.8 eV is associated with sp^2^ carbon, while other peaks are assigned to C—N/C═N and O—C═O.^[^ ^25^ ^]^ The S 2p in Figure S4e (Supporting Information) was fitted into two peaks. The peaks at 163.6 and 164.7 eV are attributed to the S 2p_3/2_ and S 2p_1/2_, respectively.^[^ ^26^, ^27^ ^]^

**Figure 2:**
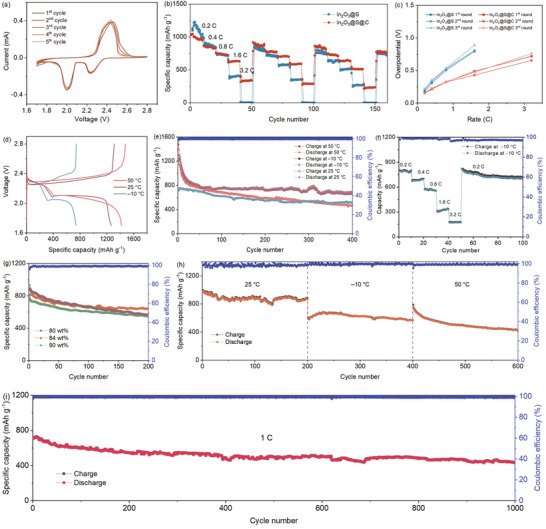
a) Initial five CV cycles of the In_2_O_3_@S@C at a scan rate of 0.1 mV s^−1^. b) Rate performance. c) Overpotentials of the discharge/charge potential plateaus at different rates. d,e) Galvanostatic charge–discharge profiles and cycling performance of In_2_O_3_@S@C at 0.2 C over 400 cycles under different temperatures. f) Capacity and Coulombic efficiency as the discharge rate was changed while the charge rate was kept at 0.2 C. g) Cycling capacity of the In_2_O_3_@S@C composite with various sulfur contents at 0.5 C under room temperature. h) Prolonged cycling behavior of In_2_O_3_@S@C with S content of 84 wt% at 0.2 C under different temperature gradients. i) Long‐term cycling performance of In_2_O_3_@S@C at 1.0 C.

The electrochemical performance of the constructed Li‐S batteries was evaluated. Samples with sulfur contents from 74.5 to 90.1 wt% were obtained based on different mass mixing and were measured by thermogravimetric analysis (TGA), as shown in Figure S5 (Supporting Information). **Figure** **2**a displays the cyclic voltammetry (CV) studies based on cathode with 74.5 wt% sulfur content in the In_2_O_3_@S@C, the CV curves show a typical cathodic peak at 2.2–2.4 V corresponds to the reduction of S_8_ to soluble long chain polysulfide (Li_2_S*_n_*, 4 ≤ *n* ≤ 8).^[^ ^28^ ^]^ The peak at 1.9–2.1 V corresponds to the process in which long Li_2_S*_n_* chain is reduced to short chain Li_2_S/Li_2_S_2_.^[^ ^29^ ^]^ The overlapped CV curves after initial scanning demonstrate high reversibility and excellent stability of redox reaction. Figure 2b and Figure S6 (Supporting Information) show the rate performance of the cathodes based on In_2_O_3_@S and In_2_O_3_@S@C during three rounds cycling from 0.2 to 3.2 C. In_2_O_3_@S@C cathode achieves highly reversible discharge capacities of 1042.9, 871.3, 759.1, 635.5, and 392.3 mAh g^−1^ at rate of 0.2, 0.4, 0.8, 1.6, and 3.2 C in first round, respectively. Even after three rounds, the capacity keeps well. In contrast, In_2_O_3_@S without carbon shell has a poor rate performance, especially at a high rate.

To quantitatively evaluate the reaction kinetics, we calculated the corresponding overpotentials at different rates of each round, as shown in Figure 2c. The In_2_O_3_@S@C cathode displays low overpotentials. Reduction of polarization is attributed to the strong chemical adsorption of polysulfides by the In_2_O_3_ core, and a powerful barrier established by the yolk–shell structure further against the irreversible diffusion of lithium polysulfides, resulting in efficient inhibition of shuttle effect. Furthermore, the carbon shell contains abundant pores which provide sufficient spaces for the absorption of polysulfides.^[^ ^30^ ^]^ The X‐ray absorption near edge structure (XANES) spectra of C K‐edge and O K‐edge (Figure S7, Supporting Information) further reveal remarkable changes of the chemical structure after cycling.^[^ ^31^ ^]^ In addition, the comparison of impedance between In_2_O_3_@S@C and In_2_O_3_@S also verifies that the carbon enhances the electron transport (Figure S8, Supporting Information). Electrochemical studies of bare In_2_O_3_ and In_2_O_3_@C without sulfur indicate that the capacity contributions from In_2_O_3_ and carbon are extremely neglectable (Figure S9, Supporting Information). Cycling at different charge versus discharge rates was also performed (Figure S10, Supporting Information). After 100 cycles, the capacities for the two cases of charge/discharge rates of 0.3 C/0.6 C and 0.6 C/0.3 C remain 922.9 and 747.7 mAh g^−1^, respectively.

The electrochemical performance of the In_2_O_3_@S@C at different temperatures was further evaluated. The first‐cycle discharge–charge profiles of the In_2_O_3_@S@C at 0.2 C under different temperatures are presented in Figure 2d. The In_2_O_3_@S@C shows an excellent cyclability at room temperature, keeping a reversible capacity of 703.3 mAh g^−1^ after 400 cycles. At the initial stage at high temperature, high‐temperature environment stimulates the internal electrochemical activity, making the In_2_O_3_@S@C exhibits high capacities (Figure 2e). As the cycling proceeds, the intensified dissolution of lithium polysulfides and the increased polarization reduce the capacity. Under a relatively low temperature, the capacity maintains a stable level, which is related to the low polarization and the effective conversion reaction of high‐order polysulfides. The low initial discharge capacity is ascribed to downward shift of the low discharging potential plateau caused by the slow solid phase deposition kinetics.^[^ ^32^ ^]^ The capacity‐contribution distribution under high and low potential plateaus reflects the changing process obviously (Figure S11, Supporting Information).

In addition, a series of cycles at different discharge rates with the same charge rate under −10 °C were measured (Figure 2f). When the cycling rates were turned back to 0.2 C, the capacity recovered to a high level, indicating the wide temperature adaptability. Figure 2g shows the specific capacities of In_2_O_3_@S@C with S contents of 80, 84, and 90 wt% at 0.5 C after 200 cycles. Moreover, the cathode with a relatively high sulfur loading of ≈4.5 mg cm^−2^ with a low electrolyte/sulfur ratio of 8 µL mg^–1^ was further investigated, which displayed a good reversible capacity, as shown in Figure S12 (Supporting Information). We also studied the prolonged cyclic performance with an 84 wt% sulfur content at different temperatures. Continuous testing was carried out at room temperature, high temperature of 50 °C and low temperature of −10 °C (Figure 2h and Figure S13, Supporting Information). In_2_O_3_@S@C remains 431.2 mAh g^−1^ after 600 cycles, which shows a good wide temperature adaptability, indicating a potential for applications. Of course, considering the price of indium, the content of In_2_O_3_ in the composite should be decreased for practical use, which could be engineered by reducing the thickness of In_2_O_3_ lamellas. Impressively, In_2_O_3_@S@C achieves a high capacity of 440.8 mAh g^−1^ even after 1000 cycles at 1.0 C with a low capacity‐fading rate of 0.038% per cycle, and the Coulombic efficiency keeps exceeding 99.5% (Figure 2i).

**Figure 3:**
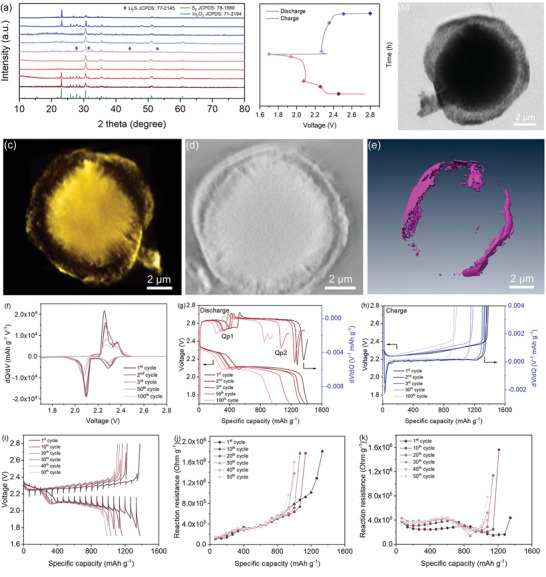
a) Ex situ XRD patterns of the In_2_O_3_@S@C when charging and discharging at various potentials. b) Synchrotron X‐ray tomography image of In_2_O_3_@C. c) 3D computed tomography image. d) Sliced image of the In_2_O_3_@C. e) 3D rendering image of carbon layer. f) d*Q*/d*V* plots (*Q*, capacity; *V*, voltage). g,h) d*V*/d*Q* versus capacity curves during discharge and charge. d*V*/d*Q* curves were obtained from the capacity test operated at a C/20 rate at 25 °C. i) Potential response curves of the In_2_O_3_@S@C electrodes during GITT measurement at room temperature with 20 min charge/discharge at 0.2 A g^−1^ followed by 1 h at rest. j,k) In situ reaction resistances of In_2_O_3_@S@C during discharge/charge processes.

In **Figure** **3**a, we conducted ex situ XRD studies on the In_2_O_3_@S@C cathode. Excepting for the S_8_ and Li_2_S reflections, no other diffraction peaks are observed, which is consistent with previous report.^[^ ^33^ ^]^ The crystal form of In_2_O_3_ with obvious sharp peak remains fixed in the process of potential change, which shows high crystallinity and stability. To further investigate a precise 3D spatial structure information, we used an X‐ray nano‐CT to reconstruct the In_2_O_3_@C. The 2D projection and 3D reconstruction images in Figure 3b,c show the obvious hierarchy of yolk–shell structure through the tomography of the hemispherical cross section of In_2_O_3_@C. Furthermore, we isolated the carbon layer by detecting the transition difference before and after the carbon absorption edge to obtain further depth distribution information (Figure 3d,e). The void between the In_2_O_3_ core and carbon layer would provide stable sites for sulfur loading. A 3D reconstruction of a sphere and a 3D rendering of the carbon layer are displayed in Movies S1 and S2 (Supporting Information), respectively. Moreover, such Li_2_S restricted at a controllable area is beneficial for continuous kinetic conversion to achieve a reversible capacity. ^[^ ^34^ ^]^

A differential potential (d*V*/d*Q*) curve obtained by differentiating the charge/discharge potential with corresponding capacity and the change of impedance during cycling, have been indicated as two indicators of the degradation of electrode.^[^ ^35^ ^]^ In our study, the capacities were measured at C/20 to minimize the contribution of the increase polarization, which were verified by d*Q*/d*V* plots (Figure 3f). The stable peak position indicates the consistency of the potential of charging and discharging plateaus. The peak shift of d*V*/d*Q* and the change in the peak‐to‐peak capacity were used to understand the capacity fade. Figure 3g,h shows the shifts in the charge and discharge curves and the d*V*/d*Q* curves of initial three cycles after 50 and 100 cycles. The slightly shift of Qp1 corresponds to effective conversion of high‐order polysulfides, the peak‐to‐peak capacity decreases with the dramatic shift of Qp2, which is consistent with the result of capacity contribution under high and low potential plateaus (Figure S11, Supporting Information). The passivation of Qp2 peak reflects the inhomogeneity caused by sluggish solid deposition reaction.

The GITT was used to in situ monitor the interfacial reaction resistances at different charge/discharge stages under various temperatures. From the first cycle, a constant current density of 0.2 A g^−1^ with 20 min charge/discharge and followed by 1 h at rest was applied to collect the potential response in every ten cycles during the GITT measurements, as presented in Figure S14a,b (Supporting Information). The interfacial reaction resistances during discharge/charge processes at room temperature always maintains a low value and a small overpotential change (Figure 3i–k). The low reaction resistance of the battery at the first cycle under high temperature corresponds to the high capacity in the initial stage; and the gradually increasing overpotential increases the interface reaction barrier and intensifies the capacity decay (Figure S14c–e, Supporting Information). At a low temperature, the initially high interface resistance reduces the reaction activity, subsequently the stable conversion of high‐efficiency polysulfides in the high potential plateau becomes the main factor for the stability of the capacity (Figure S14f–h, Supporting Information).

**Figure 4:**
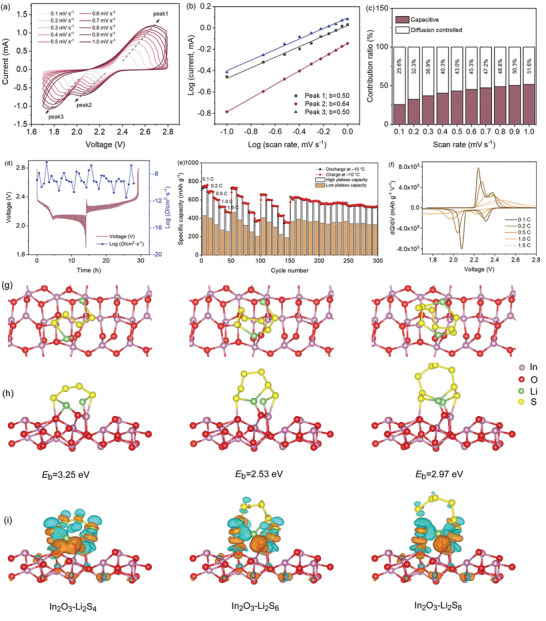
a) CV curves of the In_2_O_3_@S@C composite at a series of scan rates. b) Plots of log (*i*) versus log (*v*) and the fitting results at different redox states. c) Ratios of capacitive contributions at different rates. d) Temporal voltage evolution in GITT studies with the corresponding Li‐ion diffusion coefficients. e) Rate performance and different plateau capacity of the In_2_O_3_@S@C with S feeding ratio of 84.3 wt% cycling under −10 °C. f) d*Q*/d*V* plots of different rates. Optimized configurations of polysulfides (Li_2_S_4_, Li_2_S_6_, and Li_2_S_8_) adsorbed on In_2_O_3_ (110) surfaces: g) top view, h) side view, and i) electron density differences of corresponding structures (the gold and cyan regions represent negative and positive change of the isovalue of ±0.002).

The electrochemical kinetics were investigated by using the CV curves between 0.1 and 1.0 mV s^−1^ (**Figure** **4**a). The peak currents (*i*) and rates (*v*) are as follows: *I* = *av^b^*, and log(*i*) = *b*log(*v*) + log(*a*).^[^ ^36^ ^]^ Figure 4b shows that the *b* values for the cathodic and anodic peaks are 0.50 and 0.64, respectively, indicating a common kinetics process by diffusion‐controlled and capacitive characteristics. Based on equation *i*(*v*) = *k*_1_ *v* + *k*_2_ *v* ^1/2^, the total contribution is obtained, where *k*_1_ *v* and *k*_2_ *v* ^1/2^ represent the contributions caused by the capacitive effect and diffusion‐controlled intercalation, respectively. The ratio of the capacitive contribution increases depending on the increase of scan rates, as displayed in Figure 4c. At a scan rate of 1.0 mV s^−1^, the capacitive contribution is about 51.6%, which indicates the capacitance‐control effect under a high rate is gradually becoming significant.

The diffusion‐controlled reaction kinetics through the electrochemical process were further analyzed by using GITT. As shown in Figure 4d and Figure S15 (Supporting Information), the Li^+^ ion diffusion coefficients of the In_2_O_3_@S@C are calculated to be 3.28 × 10^−12^ to 6.26 × 10^−7^ cm^2^ s^−1^, suggesting fast Li^+^ ion diffusion kinetics.^[^ ^37^ ^]^ Moreover, several rounds of rate tests were performed with a higher sulfur content at low temperatures (Figure 4e and Figure S16, Supporting Information). By comparing the capacity contribution between high‐ and low‐plateau capacities at different current densities, it is noted that the contribution of high discharge plateau to the total capacity gradually increases in the process of increasing the current density, which is consistent with the high *b* value at the high discharge plateau. In contrast, the low discharge plateau is mainly affected by diffusion‐controlled process, which provides limited capacity due to the slow solid‐phase reaction kinetics. In addition, the cathode shows a small polarization at high rate under a low temperature (Figure 4f).

In addition, the adsorption between In_2_O_3_ and polysulfides was studied through DFT calculations. The adsorption models are displayed in Figure 4g,h. In our study, a common In_2_O_3_ facet of (110) was selected for simulations. The binding energies of polysulfides on the In_2_O_3_ are −3.2528, −2.5272, and −2.9741 eV for Li_2_S_4_, Li_2_S_6_, and Li_2_S_8_, respectively. The large adsorption energy implies that the shift of polysulfides is reduced efficiently. In Figure 4i, the bader charges analysis was further conducted to demonstrate the electron transfer. Taking the In atom as an example, the bader charges are 11.477, 11.485, and 11.461 after adsorbing Li_2_S_4_, Li_2_S_6_, and Li_2_S_8_, respectively. The brown and blue colors show the accumulation and consumption of charges. The shifts of charge verify the strong adsorption between In_2_O_3_ and polysulfides, which is able to efficiently suppress the shuttle effect of polysulfides and reduce the capacity decay caused by the loss of soluble polysulfides. Moreover, the Li_2_S_6_ solution adsorbed by In_2_O_3_@C exhibits a transparent color. It indicates the strong adsorption capability toward the polysulfides, which is confirmed by the UV–vis spectra (Figure S17, Supporting Information). The robust structure is further confirmed by the post‐cycled composite (Figure S18, Supporting Information). After cycling 1000 times, the composite keeps stable morphology and phase compared to the initial one.

## 3 Conclusions

In summary, we develop a yolk–shell In_2_O_3_@void@carbon composite as the efficient host for Li‐S battery. Owing to the lamellar inner configuration and enhanced adsorption capacity of In_2_O_3_ for polysulfides confirmed by DFT calculations, the In_2_O_3_@S@C cathode displays a good rate‐performance, long‐term cycling life with a low capacity decay rate after 1000 cycles at 1.0 C, and stable electrochemical performances in a wide temperature range. Furthermore, the rapid kinetic characteristics of continuous transformation during reversible reactions is proved by nano‐CT. The capacity decay behavior is explained by d*V*/d*Q* analysis and periodic GITT, which would provide some new insights for engineering high‐performance energy‐storage materials and their battery systems.

## 4 Experimental Section

### Synthesis of In_2_O_3_ Microspheres

The flower‐shaped microsphere precursors were fabricated by a hydrothermal strategy. Typically, 1 mmol InCl_3_·4H_2_O, 2 mmol CS(NH_2_)_2_, and 1 mmol CTAB were dissolved in 40 mL ethanol to form a uniform solution. The solution was transferred into a Teflon‐lined stainless steel autoclave and maintained at 150 °C for 12 h. And afterward, the reactor was dropped to room temperature naturally. The precipitate was collected and washed several times with absolute ethanol and deionized water. The samples were annealed in air atmosphere at 650 °C for 2 h with a ramping rate of 5 °C min^−1^.

### Preparation of In_2_O_3_@C

First of all, cover two layers of SiO_2_ on the surface of the In_2_O_3_ core. Typically, the prepared 0.2 g In_2_O_3_ was dispersed in a solution consisting of 40 mL absolute ethanol and 8 mL of deionized water by ultrasound. Then, 2 mL of ammonia aqueous solution was added under stirring. Subsequently, 1.6 mL of tetraethyl orthosilicate solution was slowly poured, and being stirred for 2 h. After standing for a few minutes, the precipitate was collected and dried. The steps shown above were repeated twice. 0.2 g of In_2_O_3_@SiO_2_ prepared above was dispersed in 50 mL of deionized water. Then, 1.211 g of tris(hydroxymethyl)aminomethane was added under stirring while hydrochloric acid was added dropwise to adjust the pH to 8.5. Subsequently, 75 mg of dopamine hydrochloride was added to the solution and stirred continuously for 24 h. 10 mL of ammonium hydroxide solution (28%–30% NH_3_) was added to 25 mL deionized water to form a dilute solution, then the In_2_O_3_@SiO_2_@C was put into the solution and transferred into a Teflon‐lined stainless steel autoclave which was then maintained at 150 °C for 8 h. Finally, the samples were calcined in nitrogen gas at 650 °C for 4 h.

### Preparation of In_2_O_3_@S@C

In_2_O_3_@C and sulfur powders were mixed in a mass ratio of 1:3 in an argon atmosphere and sealed in a heat‐resistant Teflon bottle, maintained at 155 °C for 15 h. After cooling to room temperature naturally, the In_2_O_3_@S@C was collected.

### Characterization

The morphologies, structure, and compositions of the samples were investigated on a scanning electron microscopy (FESEM, Hitachi Regulus8100, operated at 5kV) and TEM (HT‐7700). HRTEM images and the selected area electron diffraction (SAED) patterns were obtained on a JEOL JEM‐2010F microscope at an acceleration voltage of 200 kV to get the information of microstructure and crystallinity of products. The crystal structure and phase were determined by XRD (Bruker D8 Advance) with Cu Kɑ radiation at a wavelength of 1.5418 Å and XPS (ESCALAB 250Xi). Elemental analysis was measured by EDS. The surface area was measured by using BET (ASAP 2460). The specific gravity of each component was determined by TGA (LabRAM HR800) with a heating rate of 10 °C min^−1^. Raman spectra were measured by using a spectroscopy (Renishaw in Via). The ultraviolet–visible spectroscopy (U‐2910) was used to check the existence of soluble polysulfide products. XANES were carried out at the Catalysis and Surface Science Endstation at the BL11U beamline in the National Synchrotron Radiation Laboratory (NSRL) in Hefei, China. The soft X‐ray computed nanotomography was conducted on the beamline BL07W of National Synchrotron Radiation Laboratory (NSRL), Hefei, China. For the sample preparation of synchrotron‐based X‐ray nano‐CT, 10 mg of the sample powder was uniformly dispersed in 1 mL of ethanol solution by ultrasonic treatment. Then, 0.1 mL of the mixed solution was dropped on 100‐mesh, carbon‐coated copper‐finder grids (Beijing Zhongjingkeyi Technology Co., Ltd.) followed by natural drying. Finally, the grids loaded with samples were transferred into vacuum chamber for X‐ray nano‐CT tests through a transmission mode. An elliptical capillary condenser was used for the focus of the X‐ray beam. A total of 121 projections in each sample were performed at tilt angles ranging from −60° to 60° at 1° increments with the exposure to X‐ray (445 eV) within 2.0 s. For the tomographic reconstruction, the projections were reconstructed by using the total variation based simultaneous algebraic reconstruction technique (SART‐TV). All projections were corrected based on a reference image with a flat field intensity and aligned to the rotation axis. Then, the reconstructed images were imported into Amira (FEI Visualization Sciences Group, MA) for segmentation and 3D visualization.

### Electrochemical Tests

The Li‐S battery cathodes were prepared by coating the slurry of the composite (70 wt%), super P (20 wt%), and polyvinylidene fluoride binding agent (10 wt%) onto an Al foil, dried at 70 °C in a vacuum oven for 12 h. The thickness of electrode was about 250 µm. The sulfur loading and the diameter of the sulfur cathode were 2.1 mg cm^−2^ and 1.2 cm, respectively. The prepared electrodes were mounted into a coin‐cell (CR2032 type) with pure lithium foil which was used as the anode and Celgard 2400 as the separator as well as the electrolyte (1.0 m LiTFSI in a 1:1 (v/v) 1,3‐dioxolane/dimethyl ether mixture with 1.0 wt% LiNO_3_). The thickness of Li metal anode was about 500 µm, the dosage of the electrolyte used in cells were about 15 and 8 µL mg^–1^ of electrolyte/sulfur (E/S) ratio for the electrodes with sulfur loadings of 2.1 and 4.5 mg cm^−2^, respectively. All batteries were assembled in an argon‐filled glovebox (Mikrouna, Super 1220/750/900) and cycled in the potential range of 1.7–2.8 V through a galvanostatic charge/discharge method on a Neware battery test system (Shenzhen Neware Technology Co., Ltd, CT‐4008). The capacities of the coin‐type cells are calculated by the mass of the corresponding sulfur. CV curves were performed on an electrochemical workstation (CHI660E). GITT measurements were performed by discharging/charging the cell with a 20 min current pulse at 0.2 A g^−1^ followed by a 1 h rest to collect the potential response. This protocol was designed so that an equilibrium voltage (or close to equilibrium) was obtained in a reasonable experimental time. The difference between quasi‐open‐circuit potential and closed‐circuit potential is used to calculate the overpotentials, and then the in situ reaction resistance can be calculated as *R* = Δ*U*_overpotential_/(*M* ^2^_mass loading_ × *I*_current density_). The GITT tests of the diffusion coefficients of Li ions at 0.1 C with 10 min discharge–charge followed by 10 min at rest. A total of 50 current pulses was used for the discharge.

### Binding Energy Calculations

The DFT calculations were performed by using CASTEP program. The generalized gradient approximation Perdew–Burke–Ernzerh of functional was used to describe the exchange‐correlation energy. For all the calculations, the plane‐wave energy cut‐off was set to be 500 eV. For surface adsorption calculations, the Brillouin zone was sampled with 3 × 3 × 1 *k*‐point mesh. At least 12 Å vacuum layer was applied in *z*‐direction of the slab models to prevent the vertical interactions between slabs. The convergence thresholds for structural optimization were set at 0.01 eV Å^–1^ in force and 10^–5^ in energy. The binding energy between polysulfides and In_2_O_3_ surface was defined in the following equation

(1) $$E_{b}=E_{\mathrm{surface}}+E_{Li_{2}S_{n}}-E_{\mathrm{surface}+Li_{2}S_{n}}$$

where $E_{\mathrm{surface}+Li_{2}S_{n}}$ was the total energy of substrate with adsorbed polysulfides, *E*_surface_ was the total energy of substrate, and $E_{Li_{2}S_{n}}$ was the energy of polysulfides.

### Statistical Analysis

The images were processed on a Fireworks software (Adobe, San Jose, CA). The XRD, XPS, HRTEM, Raman, and electrochemical impedance spectroscopy (EIS) spectra were analyzed on Jade software (Materials Data, Livermore, CA), Peak Fit software (Systat software, Inc., San Jose, CA), Digital Micrography software (Gatan Inc, Pleasanton, CA), Labspec software (Horiba, Kyoto, JP), OMNIC (Thermo Fisher Scientific Inc., Waltham, MA), and ZView (Scribner Associates, Inc., Charlottesville, VA), respectively. The 3D synchrotron X‐ray tomography images were reconstructed on Amira software (FEI Visualization Sciences Group, MA). Results were analyzed on an OriginPro software (Origin Lab, Northampton, MA).

## Conflict of Interest

The authors declare no conflict of interest.

## Supporting information

Supporting Information

Supplemental Movie 1

Supplemental Movie 2

## Data Availability

Research data are not shared.
