# Supplementary material for: A Lamellar Yolk–Shell Lithium‐Sulfur Battery Cathode Displaying Ultralong Cycling Life, High Rate Performance, and Temperature Tolerance
Source: Adv Sci (Weinh). 2021 Nov 29;9(3):2103517. doi: 10.1002/advs.202103517 (PMC8787391; doi:10.1002/advs.202103517)
Supplement: Supplementary file 1 — Supporting Information [file ADVS-9-2103517-s001.pdf]

ADVANCED SCIENCE
Open Access

Supporting Information

for *Adv. Sci.*, DOI: 10.1002/advs.202103517

A Lamellar Yolk-Shell Lithium-Sulfur Battery Cathode Displaying Ultralong Cycling Life, High Rate-Performance and Temperature Tolerance

Jinyun Liu,[1,] Yingyi Ding,[1] Zihan Shen, Huigang Zhang, Tianli Han, Yong Guan, Yangchao Tian,[*] and Paul V. Braun*

Supporting Information

A Lamellar Yolk-Shell Lithium-Sulfur Battery Cathode Displaying Ultralong Cycling Life, High Rate-Performance and Temperature Tolerance

Jinyun Liu,[1,] Yingyi Ding,[1] Zihan Shen, Huigang Zhang, Tianli Han, Yong Guan, Yangchao Tian,* and Paul V. Braun*

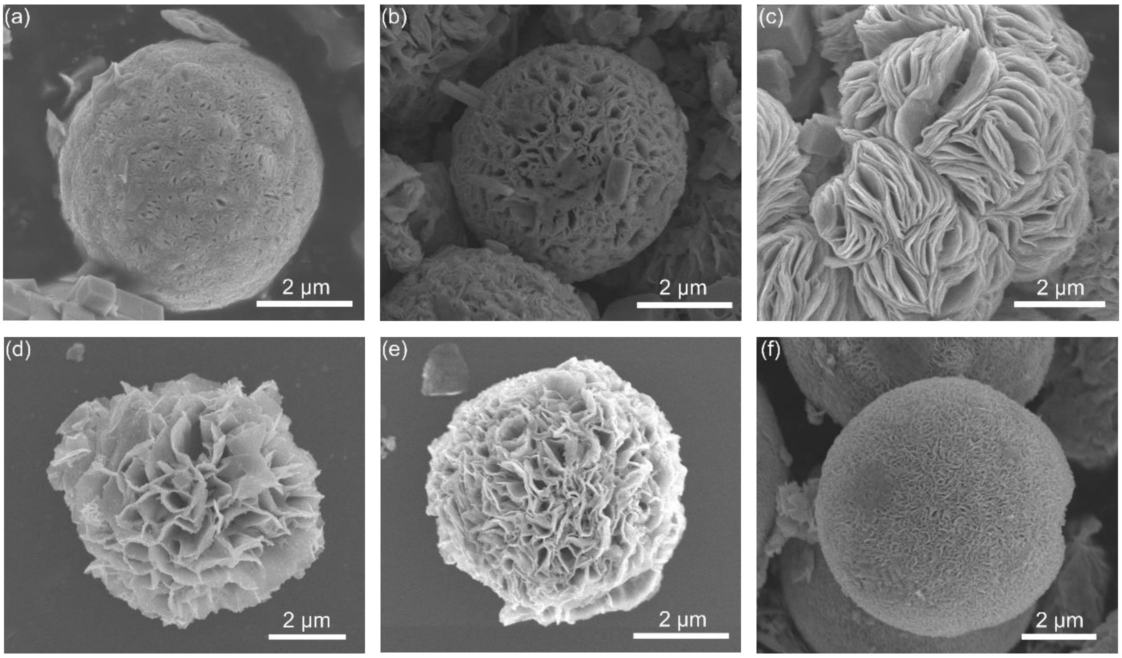

Figure S1. SEM images of the In_2O_3 prepared under different conditions: (a) 0.5 mmol, (b) 1.5 mmol and (c) 2.0 mmol CTAB at 150 ºC for 12 h; 1 mmol CTAB at (d) 140 ºC, (e) 150 ºC and (f) 160 ºC for 12 h.

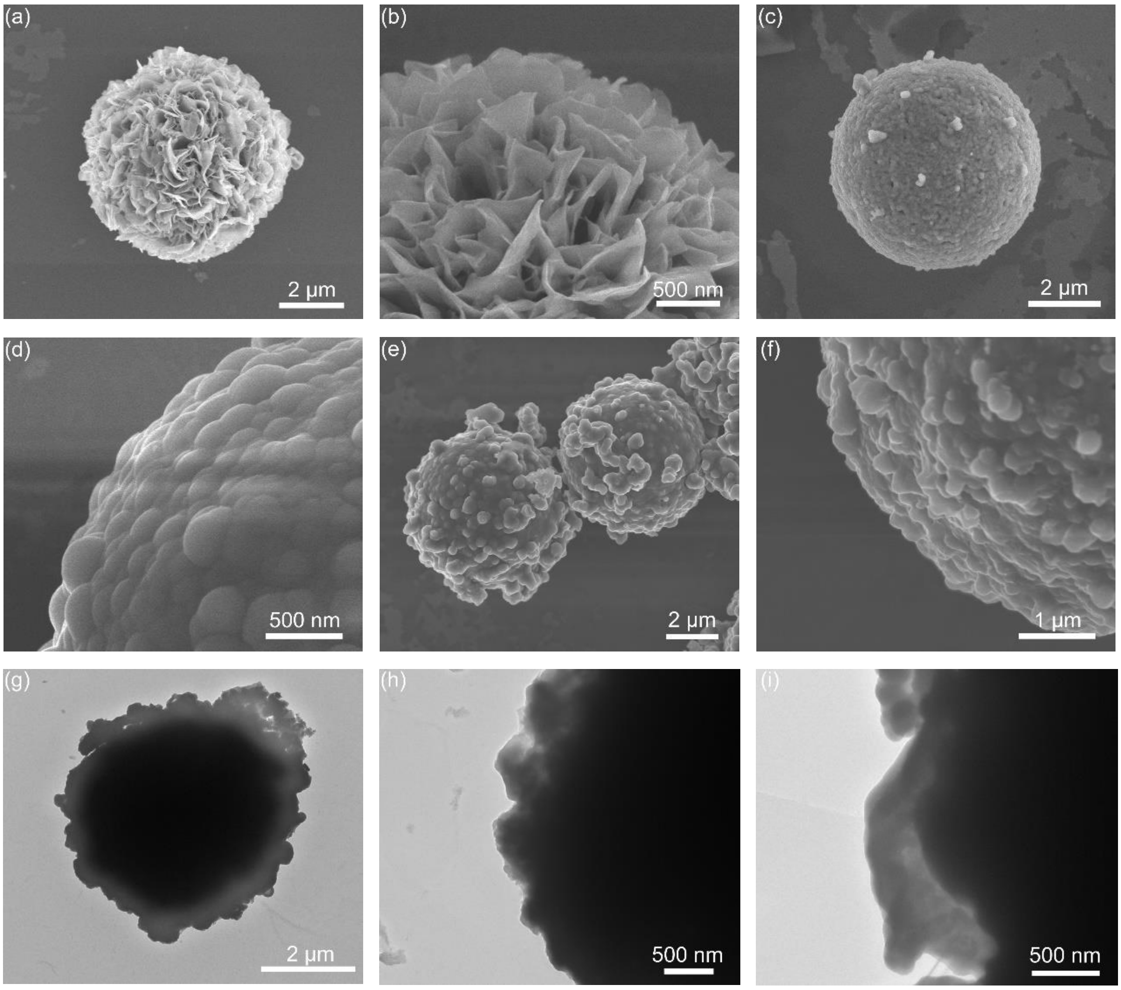

Figure S2. SEM images of (a,b) In_2O_3, (c,d) $In_2O_3@SiO_2$ and (e,f) $In_2O_3@S@C$ in different scale. TEM images of (g) $In_2O_3@C$ and (h,i) edge magnification image of $In_2O_3@C$.

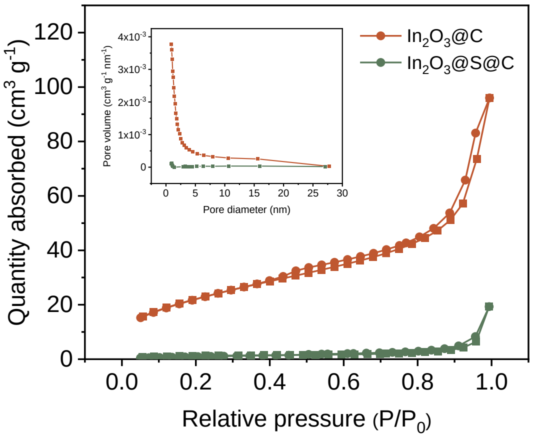

Figure S3. N_2 adsorption-desorption isotherms inserted with corresponding pore-size distribution profile of the $In_2O_3@C$ and $In_2O_3@S@C$.

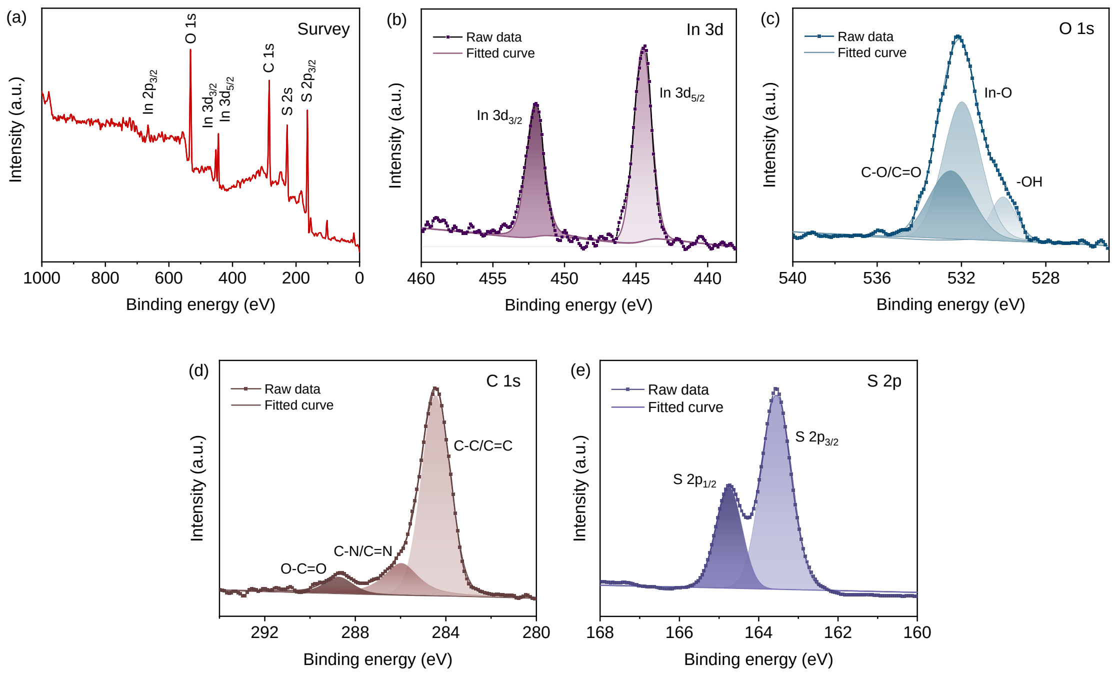

Figure S4. XPS spectra of the In_2O_3@S@C: (a) survey spectrum, (b) In 3d, (c) O 1s, (d) C 1s, and (e) S 2p.

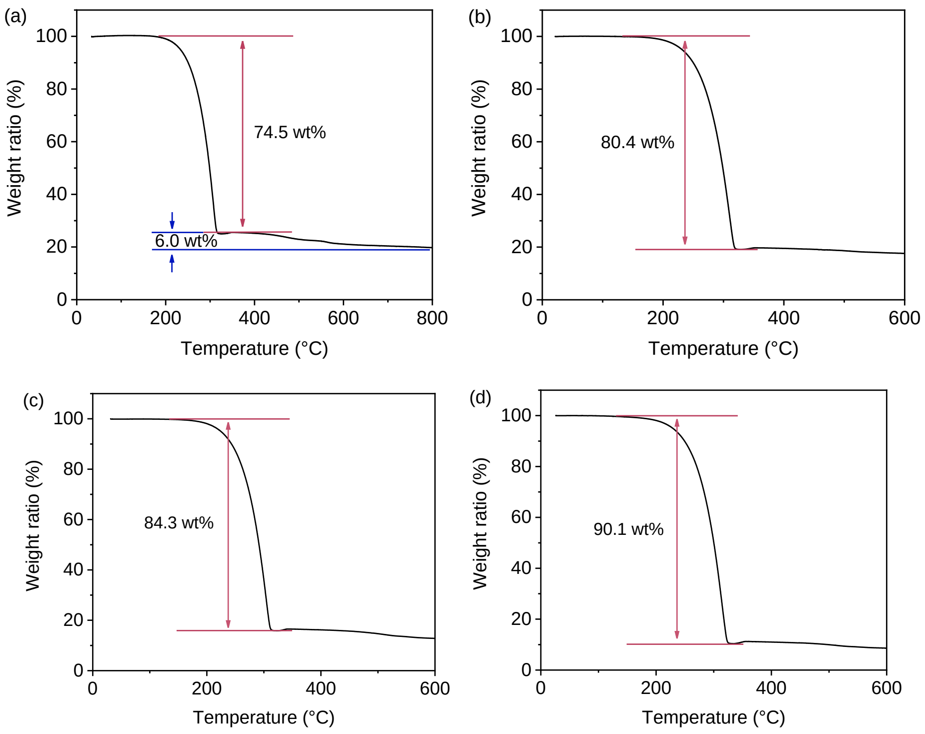

Figure S5. The thermogravimetry (TG) curve of In_2O_3@S@C with S feeding ratio of (a) 74.5 wt%, (b) 80.4 wt%, (c) 84.3 wt% and (d) 90.1 wt%.

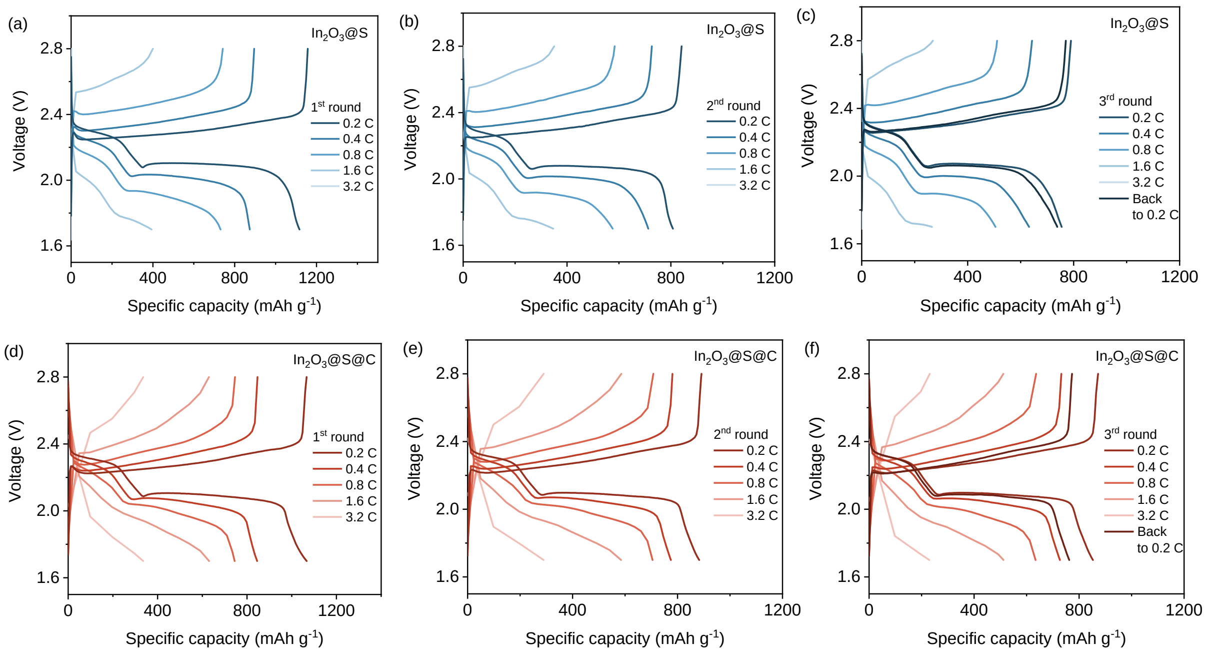

Figure S6. The galvanostatic discharge–charge profiles of the electrodes with S feeding ratio of 74.5 wt% at different rates. (a-c) The discharge–charge profiles of In_2O_3@S and (d-f) In_2O_3@S@C.

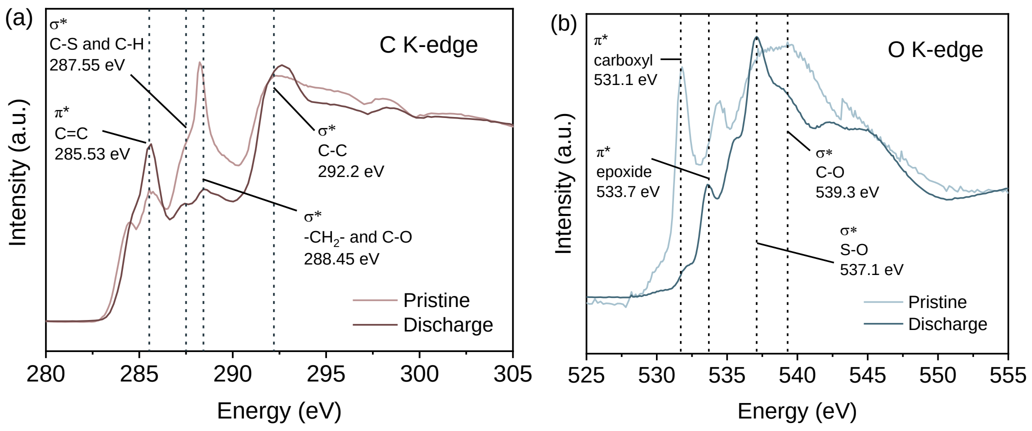

Figure S7. (a) C and (b) O K edge XANES spectra of the In_2O_3@S@C.

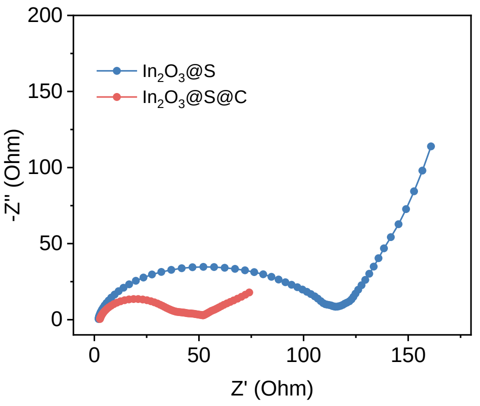

Figure S8. The EIS spectra of In_2O_3@S@C and In_2O_3@S.

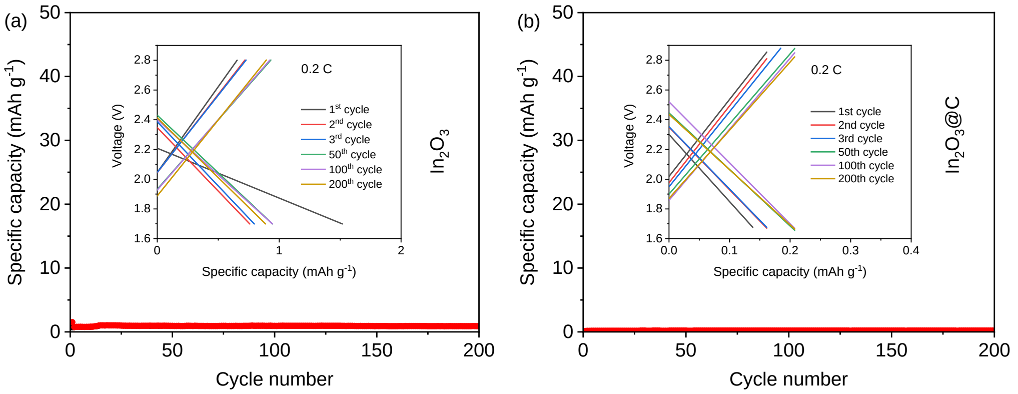

Figure S9. The electrochemical performance of In_2O_3 and In_2O_3@C compounds without sulfur.

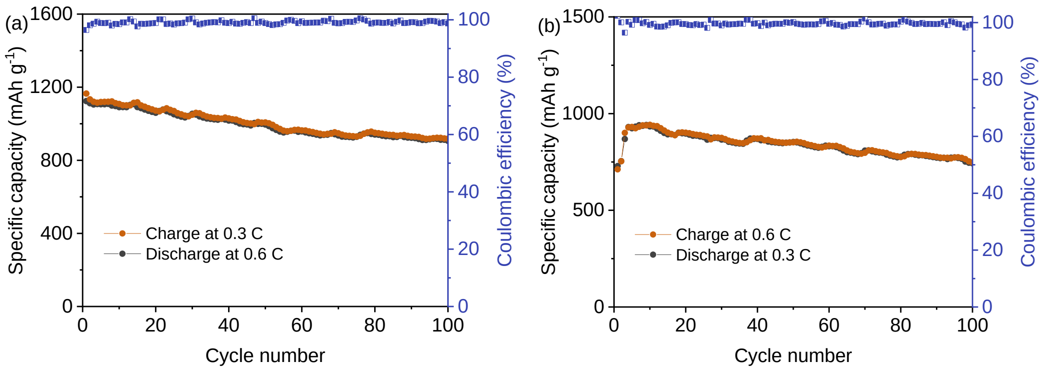

Figure S10. Cycling performance of the In_2O_3@S@C composite with S feeding ratio of 74.5 wt% at (a) charge rate of 0.3 C vs. discharge rate of 0.6 C, and (b) charge rate of 0.6 C vs. discharge rate of 0.3 C.

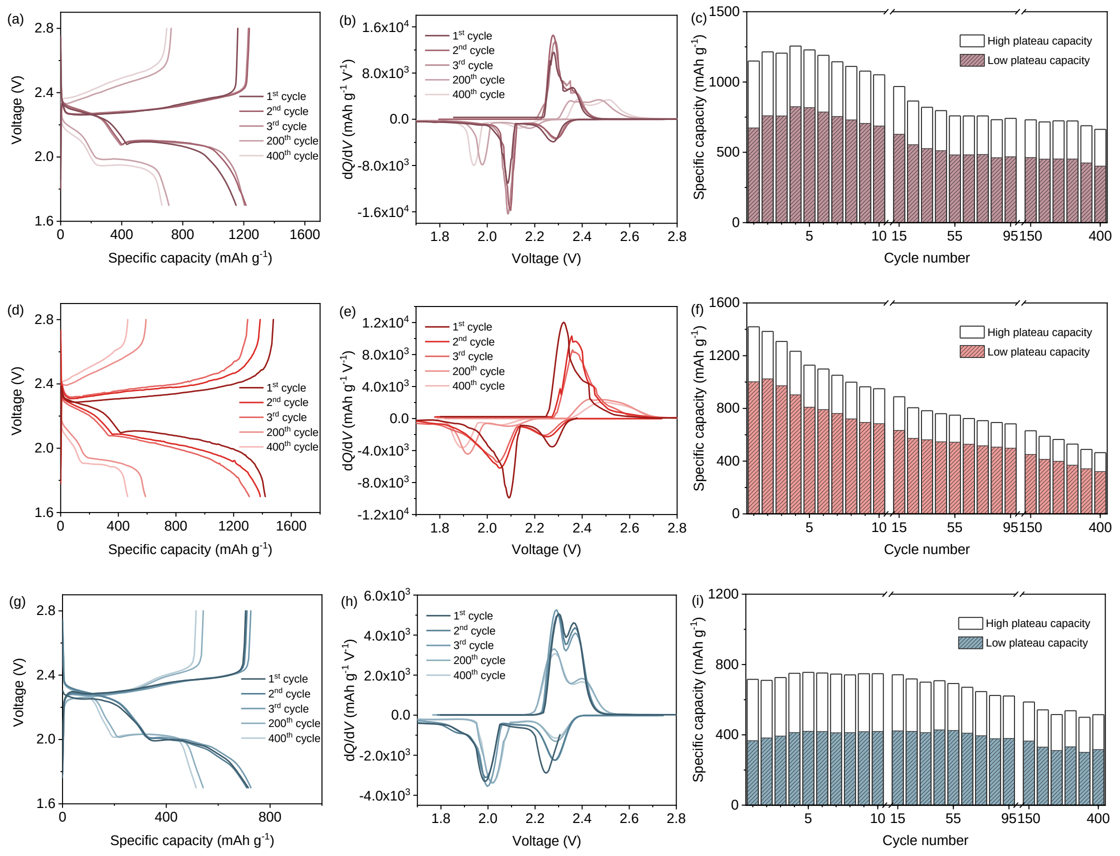

Figure S11. (a-c) Discharge–charge profiles, dQ/dV plots and different plateau capacity of the In_2O_3@S@C with S content of 74.5 wt% cycling at 0.2 C under 25 °C, (d-f) cycling under 50 °C, (g-i) cycling under –10 °C.

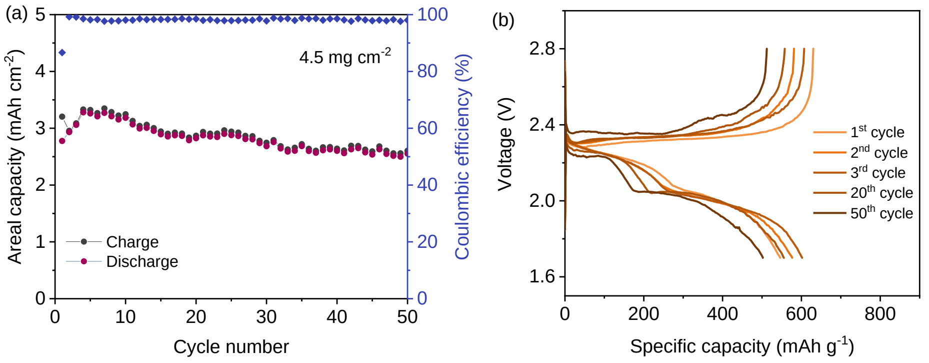

Figure S12. (a) Cycling performance and Coulombic efficiency at 0.1 C for 50 cycles with a sulfur loading of 4.5 mg cm^{-2} and (b) galvanostatic charge-discharge profiles at 0.1 C.

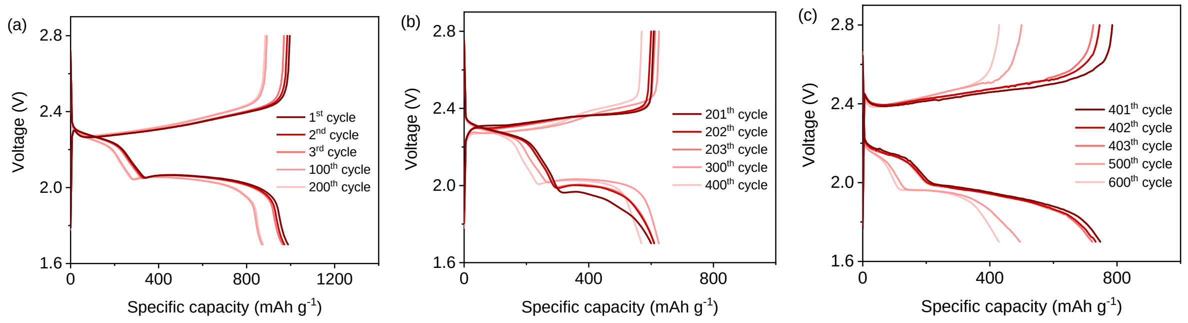

Figure S13. The galvanostatic discharge and charge curves of the In_2O_3@S@C with S feeding ratio of 84.3 wt% cycling at 0.2 C under different ambient temperature, (a) 25 °C, (b) –10 °C, (c) 50 °C.

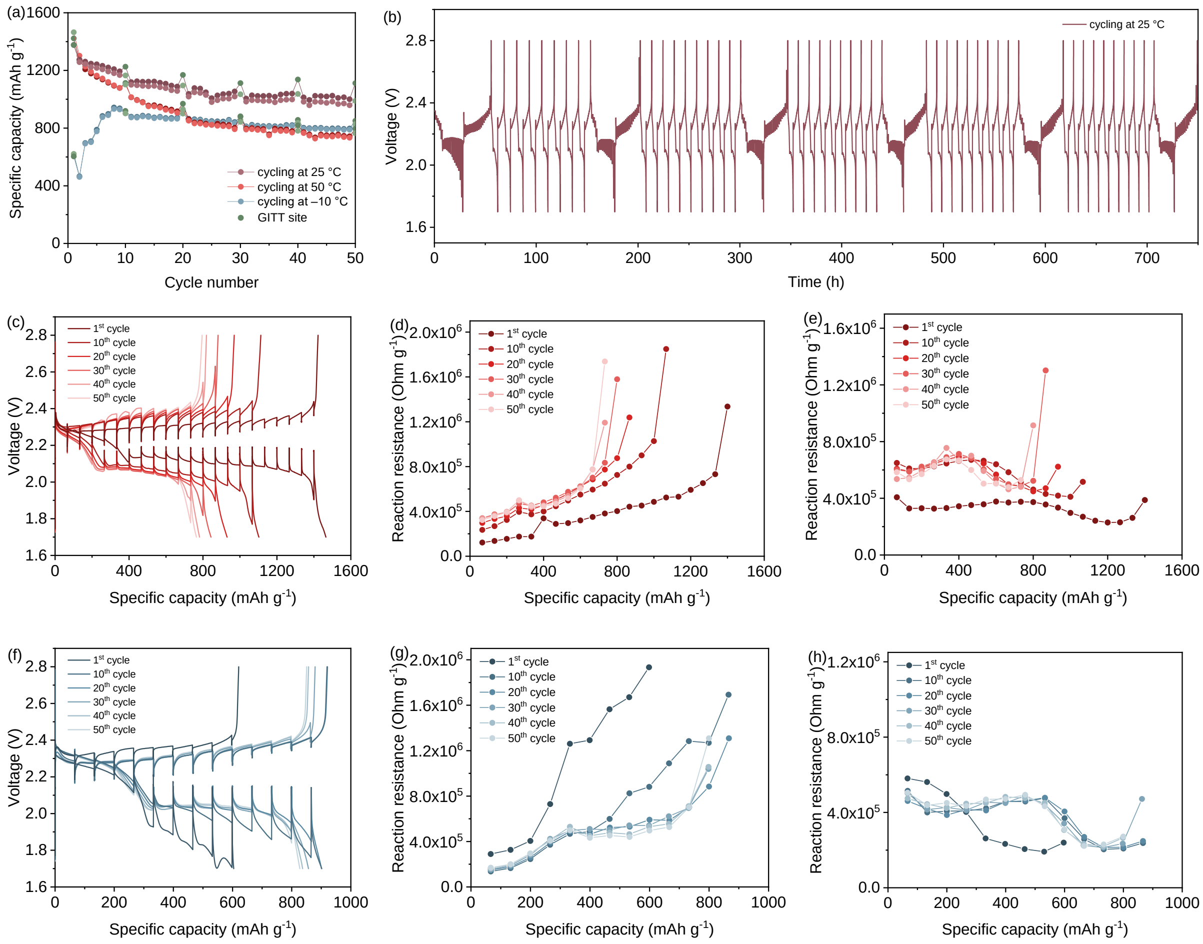

Figure S14. (a) Cycling capacity of the In_2O_3@S@C composite with GITT measurement at current density of 0.2 A g^{-1} under various temperature. (b) Voltage vs. time profile at 25 °C. (c) The potential response curves of the In_2O_3@S@C electrodes during GITT measurement at 50 °C. (d,e) *In situ* reaction resistances of In_2O_3@S@C during discharge/charge processes at 50 °C. (f-h) cycling under –10 °C.

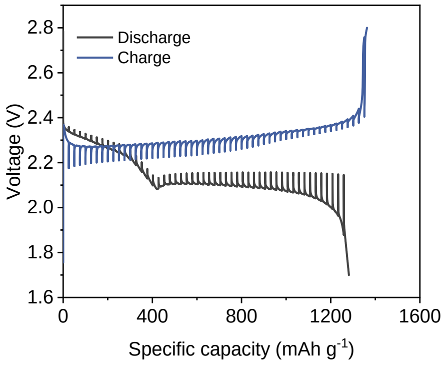

Figure S15. The potential response curves of the In_2O_3@S@C electrodes during GITT measurement, with 10 min charge/discharge at 0.1 C followed by 10 min at rest.

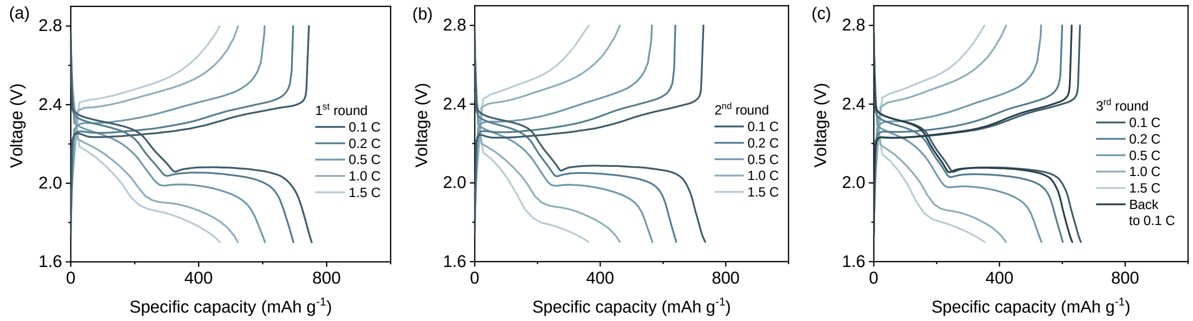

Figure S16. The discharge and charge profiles of the In_2O_3@S@C with S feeding ratio of 84.3 wt% at the (a) first, (b) second, and (c) third round of rate-performance measurements cycling under –10 °C.

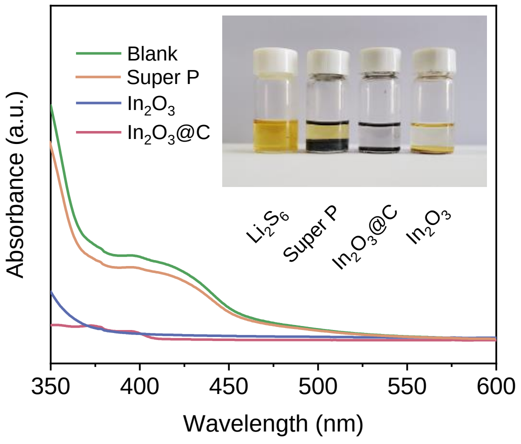

Figure S17. The absorbance profiles of the Li_2S_6 electrolytes before and after adding the In_2O_3 and In_2O_3@C as the absorbents, respectively. The inset is the pictures of the electrolytes after absorption tests.

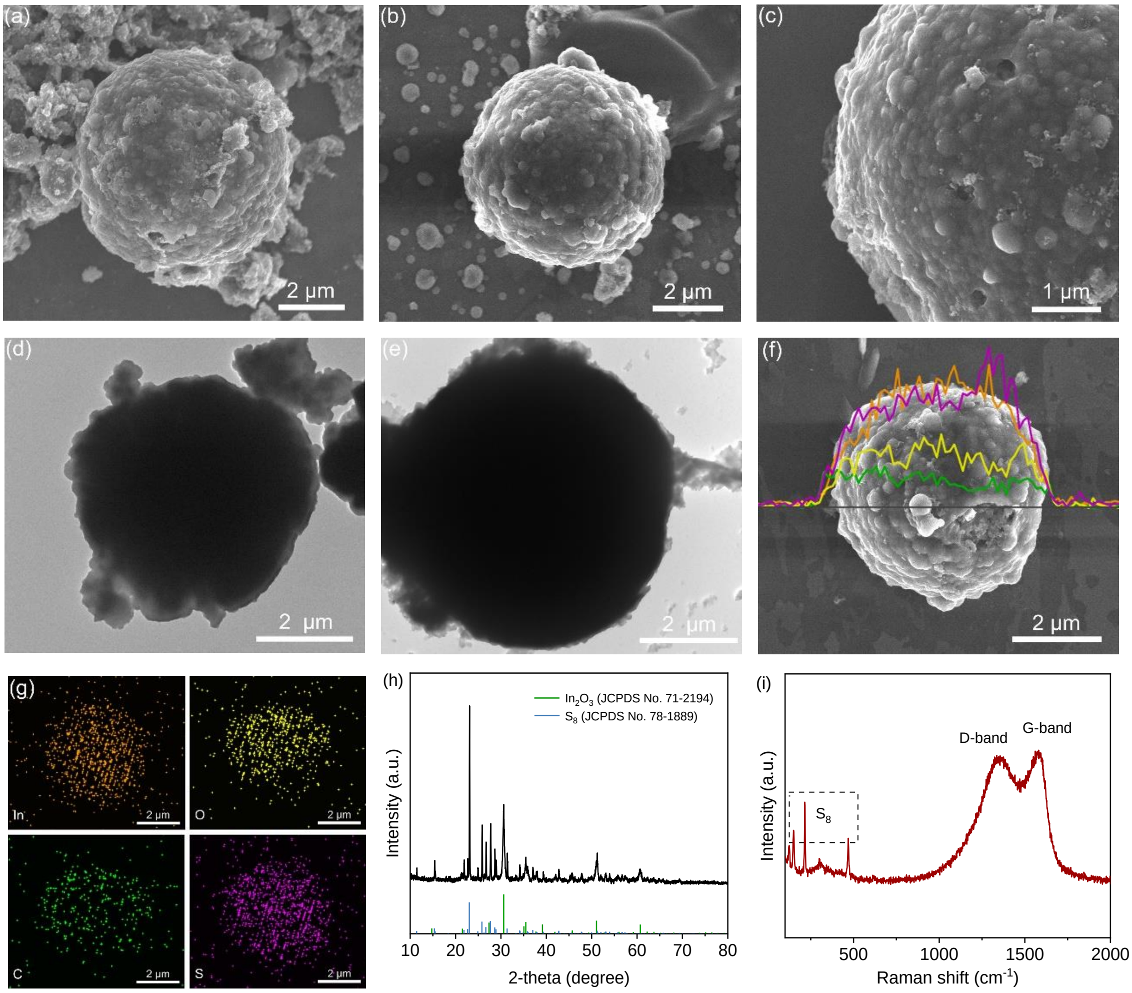

Figure S18. (a-c) SEM and (d,e) TEM images of the In_2O_3@S@C composite after cycling 1000 times at 1.0 C. (f,g) Line-scanning curves and elemental mapping images of the In_2O_3@S@C composites after cycling. (h,i) XRD pattern and Raman spectra.
